# Spatially guided translation from histology images to transcriptomic profiles using foundation model-driven contrastive learning

**DOI:** 10.1371/journal.pcbi.1014762

**Published:** 2026-09-08

**Authors:** Zi Huai Huang, Ziyang Xu, Pingzhao Hu

**Affiliations:** 1 Department of Biochemistry, Western University, London, Canada; 2 Department of Computer Sciences, Western University, London, Canada; 3 Division of Biostatistics, University of Toronto, Toronto, Canada; The Chinese University of Hong Kong Faculty of Science, HONG KONG

## Abstract

Spatial transcriptomics (ST) enhances single-cell RNA sequencing by revealing transcript distribution, offering critical insights into heterogeneous diseases such as breast cancer. However, the high cost and lengthy processes of generating high-quality ST data limit clinical application. Recent deep learning methods predict ST from histology images, but often fail to capture both morphological features and spatial context. We introduce FOCST, a foundation model-driven framework for ST imputation that leverages spatial guided contrastive learning. FOCST begins with UNI, a large histopathology foundation model, to extract visual features from tissue images. These are integrated with expression data in a unified embedding space via contrastive learning, enabling cross-modal prediction and imputation. To further enhance spatial awareness, a graph neural network incorporates positional information, improving regional detection and interpretability.Benchmarking demonstrates FOCST’s superior performance over state-of-the-art methods and alternative vision encoders (paired Wilcoxon signed-rank tests, FDR-adjusted p < 0.05, N = 6 images). Predicted profiles enable clinically relevant downstream analyses, including patient stratification by treatment response (ROC AUC (Receiver Operating Characteristic – Area Under the Curve) = 0.79). Our results highlight the promise of combining foundation models and spatially guided learning to efficiently generate ST insights, advancing cancer research and precision medicine.

## Author summary

Spatial transcriptomics offers powerful insights into tissue heterogeneity but remains expensive and impractical for large-scale clinical use. We developed FOCST, a foundation model-based approach that predicts transcriptomic profiles from standard histology images using spatially guided contrastive learning. By leveraging UNI, a large histopathology model, and a graph neural network to encode spatial context, FOCST produces highly accurate and interpretable ST predictions. Compared to existing methods, it achieves superior accuracy and supports downstream analyses such as patient treatment response prediction. FOCST demonstrates the potential of foundation models to bring cost-effective, scalable spatial biology into cancer research and clinical translation.

## Introduction

Spatial transcriptomics (ST) has emerged as an extension of single-cell RNA sequencing, offering valuable insights into the spatial distribution of RNA transcripts [1]. An example underscoring the significance of ST data and its applications can be observed in the context of the heterogeneous disease HER2 + breast cancer (BC) [2]. Within these BC tissues, cells engage in active interactions with the surrounding tumor microenvironment, effectively subverting immune defense mechanisms and promoting proliferation [3,4]. Therefore, capturing spatial differences in gene expression will provide more insight into the disease and patient outcomes [5]. ST technology has evolved rapidly over recent years, from detecting only a few genes (seqFISH, MERFISH) to hundreds of thousands and currently, at the whole transcriptome level (seqFISH+), while incorporating the power of next generation sequencing (NGS) to achieve higher sample throughput [6–8]. Current ST technology is generated based on spatial barcoding followed by next generation sequencing (NGS), where transcriptome-wide gene expression is measured in gene capture locations, referred to as spatially barcoded spots. These spatial barcoding-based ST technologies, such as 10X genomics Visium, SLIDE-seq, and SLIDE-seq2, are all accompanied by a high-resolution hematoxylin and eosin (H&E) stained histology image of the tissue section from which the gene expression data was obtained [9–11]. However, despite massive advancements in ST technology, the high cost needed to generate such data poses a major limitation to its applicability to large-scale studies. Relatively, the histology images that are provided alongside ST data are routinely used in clinical practice and are much less expensive to obtain.

This potential gave rise to a new avenue of research that is focused on leveraging various deep-learning models to predict ST data directly from cost-effective histology images, which are often paired with ST datasets. The synthesis of ST data through this approach offers significant advantages, including cost-effectiveness, accelerated data acquisition, and enhanced data availability. As a result, this allows for larger scale investigations regarding spatial differences in gene expression [12].

Several studies have provided validation for the feasibility of generating ST data from histology images, including HE2RNA [13], ST-Net [14], HisToGene [15], hist2rna [16], Hist2ST [17], BrST-net [12], BLEEP [18] and other methods [19,20]. Among these existing methods, ST-Net predicts spatially variable gene expression across whole histology images by segmenting the image into patches and individually predicting each patch using DenseNet [21]. Although effective, ST-Net is limited by its independent patch-level predictions, which neglect spatial context and interactions between adjacent tissue spots. HisToGene was then introduced by Pang et al., which leverages spot spatial relations through a Vision Transformer (VIT) [15,22]. This attention-based VIT model effectively models the global positional relationships among spots by incorporating positional embeddings. However, HisToGene fails to model the interactions between local image patches which often provide crucial information in the context of tumor micro-environments. Hist2ST expands further upon this approach by employing a combination of convolutional modules, Transformer modules, and graph neural networks, achieving improved results [17]. Although Hist2ST considers modeling both local and global relationships, the positional encoding utilized is limited to the absolute coordinate embeddings and are simple representations that do not capture the more nuanced relative spatial structures or global tissue connectivity patterns that are present in cancer tissues. Considering that image features cannot quantify the expression of all genes, and the gene panel used for prediction is limited, the BLEEP model is based on a contrastive learning framework and predicts gene expression using a reference dataset [18]. However, it also lacks spatial positional encoding and limits its ability to capture the spatial organization of gene expression patterns that are critical in tumor microenvironments.

While existing methods show promise in ST prediction, they often fall short in capturing the intricate relationship between the detailed morphological features of each image patch, the local neighboring relationship between patches, and the global spatial relationship between all image patches and their expression patterns [23,24]. Additionally, predicted ST data has yet to be fully validated for clinical viability in downstream analyses involving unpaired BC patients. More recently, THItoGene was proposed to address the challenge of ST generation as well [25]. It employs dynamic convolution and capsule networks to adaptively capture multiscale cellular features and integrates positional information by encoding spot coordinates into embeddings that are fused with image features via a ViT. Additionally, it uses Graph Attention Networks (GAT) to model spatial relationships on a k-nearest neighbor graph. While THItoGene represents a significant advance in spatial feature modeling, it relies on custom convolutional architectures that lack access to the powerful pretrained representations available from foundation models. Furthermore, its positional encoding approach treats spatial coordinates as simple numerical embeddings rather than explicitly encoding the graph structure of tissue neighborhoods, and it uses a direct reconstruction objective rather than a contrastive learning framework that can leverage reference datasets for more robust cross-sample inference.

Motivated by these limitations, we present FOCST (Foundation model-based Contrastive Learning for Spatial Transcriptomics), a novel framework designed to predict spatial transcriptomics (ST) profiles from histology images. (1) Leveraging recent advancements in computational pathology and self-supervised learning, FOCST employs UNI, a state-of-the-art pathology foundation model pretrained on extensive histopathology datasets (the Genotype-Tissue Expression Consortium and Mass-100k dataset containing over 100 million tissue patches across 20 tissue types), as a powerful vision encoder. The incorporation of the foundation model should enhance generalizability of the model to different tissue and cancer types. This robust foundation model significantly improves visual feature extraction, surpassing previous CNN or standard vision transformer-based methods. (2) To jointly model morphological image features and spatially resolved gene expression data, we integrate this foundation model with Graph Neural Networks (GNNs), creating a shared embedding space. This contrastive approach explicitly aligns visual representations from image patches with corresponding gene expression profiles, leveraging a reference dataset to enhance prediction stability and accuracy. (3) Critically addressing previous limitations in modeling global spatial dependencies, we introduce an innovative transformer-based autoencoder module coupled with a GAT-derived positional encoding. Unlike prior methods such as Hist2ST that relied primarily on absolute coordinates for spatial encoding, FOCST explicitly incorporates spatial neighborhood context, capturing both local interactions and complex global spatial relationships among tissue regions. We demonstrate that FOCST achieves state-of-the-art performance in gene expression predictions. Importantly, the predicted spatial expression profiles carry biological significance, making them valuable for downstream analyses and clinical application for cancer research. We organize the remainder of the paper as follows: Methods (datasets, architecture, and evaluation protocols), Results (benchmarking, ablation studies, gene visualization, and clinical validation), Discussion (interpretation and future directions), and the Conclusion.

## Methods

### Datasets and preprocessing procedures

#### Datasets.

We utilized a HER2-positive breast cancer, a cutaneous squamous cell carcinoma (cSCC) ST dataset [17], and a lung cancer dataset [26]. The HER2-positive dataset comprises 36 tissue sections from 8 patients, featuring their histology images, spatially resolved gene expressions, and corresponding spot coordinates [4]. While the cSCC dataset is derived using 10X Visium technology and features 4 patients with 3 tissue sections each [17]. Lastly, the lung cancer dataset is also derived using 10X Visium technology and features 5 tissue sections from 1 patient.

#### Image preprocessing.

For histological images, we extracted image patches of size W × H around each sequencing spot, where W and H are set to 224, corresponding to the diameter of each sequenced spot. The patch size was chosen to match the physical diameter of sequenced spots and to ensure that there is only one patch per sequencing location. No patch overlap was applied, following the native spatial layout of sequenced ST spots.

Stain normalization was not applied to the HER2-positive, cSCC, and Lung datasets, as they all had consistent staining protocols. However, for the Yale trastuzumab response cohort (Treatment Response Prediction section), we applied stain normalization due to inconsistencies in slide preparation.

#### Gene expression and spatial graph construction.

We apply global gene filtering where the top 1000 HVGs were selected and genes that are expressed in less than 1,000 spots were excluded. This filtering strategy is consistent with established protocols from Hist2ST and THItoGene and ensures the removal of sparse features. For the coordinates in the ST data, we calculated the Euclidean distance between each spot u and every other spot v. We then identified the k = 4 nearest points $v_{\text{1}},v_{\text{2}},\ldots ,v_{k}$ as adjacent nodes in the graph, creating an edge set E between all nodes v. Finally, we compute the edge weights $w(u,v)=\text{exp}\left(-\frac{d(u,v{)}^{\text{2}}}{\text{2}}\right)$ based on the negative exponential function of the distance, resulting in the adjacency matrix A of sequencing points. Importantly, the raw count data was preserved without log-transformation or library size normalization to maintain compatibility with the ZINB distribution assumption in our loss function.

After preprocessing, the HER2-positive dataset retained 785 genes and 13,620 spots in total across 36 images, the cSCC dataset retained 8671 spots with 171 genes, and the lung dataset retained 16,032 spots with 415 genes. We adopted a data-splitting strategy distinct from prior studies. The training set was fixed, while the test set comprised of images from patients not included during training. For the HER2-positive dataset, 30 images containing 10,523 total spots were allocated for training, while 3 images from 2 independent patients, encompassing 3097 total spots, were reserved for testing. The cSCC dataset, 9 images with 6980 spots were used for training, and 3 images from a single patient were designated for testing and lastly, the lung data comprised of 4 images with 14,015 for training and 1 image with 2017 spots for testing. (Table 1).

**Table 1:** Dataset used for FOCST training and testing.

Datasets	Types	Number of Patients	Number of Genes	Number of Images	Number of Patches
HER2+	Training set	6	785	30	10523
HER2+	Test set	2	785	6	3097
cSCC	Training set	3	171	9	6980
cSCC	Test set	1	171	3	1691
Lung	Training set	1	415	4	14015
Lung	Test set	1	415	1	2017

### Image and expression encoders

**Fig 1:**
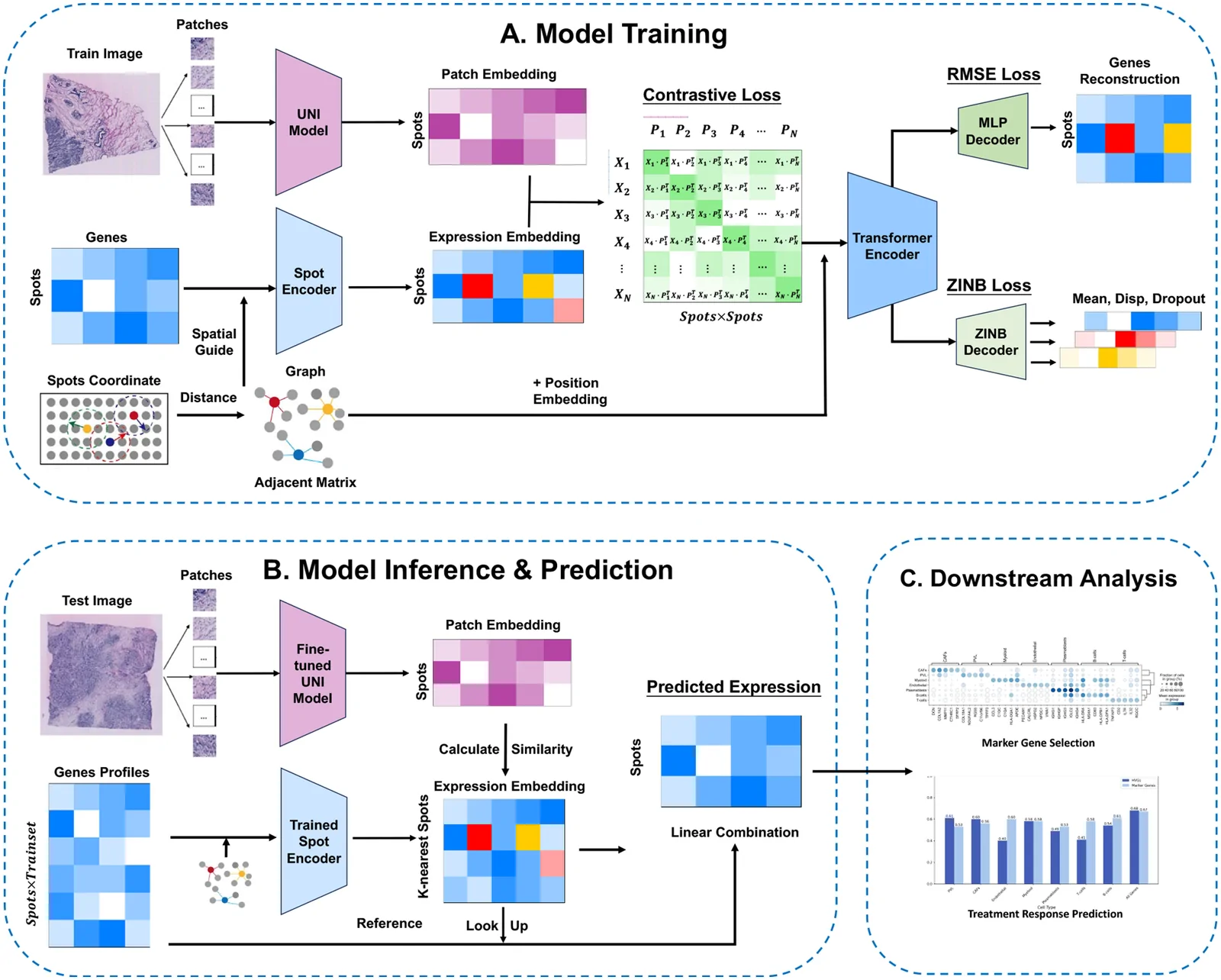
The Architecture of FOCST. FOCST consists of three main components: Model Training, Model Inference & Prediction, and Downstream Analysis. During data preprocessing, histology images are cropped around each sequencing spot into individual image patches. **A**. Model Training: Cropped image patches and their corresponding gene expression profiles are fed into the UNI model and a graph convolutional encoder to extract 2D visual features and spatial features, respectively. The extracted image and gene expression features are then aligned into a shared space using separate projection heads to calculate the contrastive loss. Additionally, gene expression embeddings are imputed using a Transformer-based autoencoder, with reconstruction loss computed using RMSE and the ZINB distribution. The final loss is a weighted sum of all the computed losses. **B.** Model Inference & Prediction: Gene expression for query image patches is predicted within the contrastive learning framework by imputing expression profiles from a reference dataset. **C.** Downstream Analysis: The predicted ST profiles undergo further downstream analysis to validate their clinical relevance for HER2-positive breast cancer patients.

In the first step of our model (Fig 1), we implement the histopathology foundation model, UNI, as our image encoder to process the image patch denoted as P∈ $R^{N\times C\times W\times H}$, Where $N\;=n_{\text{spot }}$represents the number of sequencing spots in an image patch and C×W×H=3×224×224 corresponds to the channel size and dimensions of each patch. UNI then extracts features from each patch into a feature vector with ${dim}_{\text{img }}=\text{7}\text{8}\text{5}$/171/415. The output of UNI can be denoted as $P_{\text{feat }}=\text{UNI}(P)\in R^{N\times {dim}_{img}\;}$.

For gene expression, denoted as ${\text{X}}_{\text{feat }}\in R^{\text{N}\times \text{di}{\text{m}}_{\text{spot}}}$, it is integrated with the adjacency matrix $A\in R^{N\times N}$ to extract spatial features utilizing Graph Convolutional Networks (GCN) [27]. Here, $N=n_{\text{spot }}$ represents the number of spots in each tissue slice, ranging from 600 - 1100. $n_{\text{gene }}$indicates the total number of genes to be predicted, where for the HER2-positive dataset, $n_{\text{gene }}=\text{7}\text{8}\text{5}$, the cSCC dataset, $n_{\text{gene }}=\text{1}\text{7}\text{1}$, and for the lung dataset $n_{\text{gene }}=\text{4}\text{1}\text{5}$.

By leveraging the message-passing framework of GCNs, each node’s features are updated based on information from its neighboring nodes, enabling the model to capture local spatial relationships and gene expression patterns. This process allows the GCN to learn spatially aware representations that are critical for accurate downstream predictions. The updated framework is defined as:


$$\begin{array}{c} \begin{array}{c} {\text{H}}^{\left(l+\text{1}\right)}=\sigma \left(LN\left({\tilde{D}}^{-\frac{\text{1}}{\text{2}}}\tilde{A}{\tilde{D}}^{-\frac{\text{1}}{\text{2}}}H^{\left(l\right)}{\text{W}}^{\left(\text{1}\right)}\right)\right) \end{array} \end{array}$$ (1)

where ${\text{H}}^{\left(\text{l}\right)}$ is the feature matrix at layer l, $\tilde{\text{A}}=\text{A}+\text{I}$ is the adjacency matrix A with added self-connections, $\tilde{D}$ is the degree matrix of $\tilde{A}$, ${\text{ W}}^{\left(l\right)}$ is the weight matrix for layer l, LN is layer normalization, and σ represents the ReLU activation function. We set the number of GCN layers L=3, the hidden channels ${\text{dim}}_{\text{spot }}=n_{\text{gene }}$. Specifically, the input ${\text{H}}^{\left(\text{0}\right)}=\text{X}\in$ $R^{n_{\text{spot }}\times n_{\text{gene }}}$and the output ${\text{X}}_{\text{feat }}={\text{H}}^{\left(\text{L}\right)}\in R^{\text{N}\times {\text{dim}}_{\text{spot }}}$.

### Projection embeddings and contrastive loss

The image features ${\text{P}}_{\text{feat }}\in R^{\text{N}\times \text{di}{\text{m}}_{\text{img}}}\;$and the gene expression features ${\text{X}}_{\text{feat }}\in R^{\text{N}\times \text{di}{\text{m}}_{\text{spot}}}$ generated in the preceding step are subsequently aligned into a unified 256-dimensional space through specialized projection heads, mainly composed of linear layers. These projection heads, composed mainly of linear layers, create embeddings for both the image patches and gene expressions, preparing them for contrastive loss computation. The resulting embeddings are denoted as, ${\text{P}}_{\text{emb}}\in R^{\text{N}\times {\text{dim}}_{\text{proj}}}$ and ${\text{X}}_{\text{emb}}\in$ $R^{\text{N}\times \text{di}{\text{m}}_{\text{proj }}}$, where ${\text{dim}}_{\text{proj }}=\text{256 }$is the projection dimension.

Inspired by CLIP we utilize a contrastive learning loss to optimize the alignment between image and gene expression features. For a batch containing N (image, gene expression) pairs, the contrastive learning component in FOCST aims to predict which one of the N×N possible (patch, gene expression) pairings within a batch occurred. To achieve this, FOCST maps the image and gene expression features into a shared multi-modal embedding space, maximizing the cosine similarity of N correct pairs while minimizing the similarity of $N^{\text{2}}-N$ incorrect pairings.

Unlike images in CLIP, FOCST processes an entire tissue histology image, dividing into N patches, which are input into the model as a batch. This leads to the sampling of multiple spots with similar expression profiles or image morphologies within the same batch. To prevent the model from incorrectly separating spots with similar expression profiles, we incorporate a smoothing scheme into the contrastive loss computation. Specifically, we first generate paired similarities through $\left({\text{P}}_{\text{emb}},{\text{X}}_{\text{emb}}\right)={\text{X}}_{\text{emb}}\cdot {\text{P}}_{\text{emb}}^{\text{T}}$. To account for pairs with similar morphological features or expression profiles, we start with calculating internal similarities of ${\text{P}}_{\text{emb}}{\text{P}}_{\text{emb}}^{\text{T}}$ and ${\text{X}}_{\text{emb}}{\text{X}}_{\text{emb}}^{\text{T}}$. The final adjusted similarity target matrix is denoted as:


$$\begin{array}{c} \begin{array}{c} \text{ target }=\text{softmax}\left(\frac{P_{emb}P_{emb}^{T}+X_{emb}X_{emb}^{T}}{\text{2}\tau }\right) \end{array} \end{array}$$ (2)

Where, τ = 1.0 is a temperature hyperparameter. Ultimately, cross-entropy (CE) loss is used to align the image and gene expression embeddings, and the final contrastive loss $L_{\text{cont }}$computed as:


$$\begin{array}{c} \begin{array}{c} \;L_{\text{cont }}=\text{mean}(\text{CE}({\text{X}}_{\text{emb }}\cdot {\text{P}}_{\text{emb }}^{\text{T}}\;\;\;\;\;\;,\text{ target})+\text{CE}({\text{P}}_{\text{emb }}\cdot {\text{X}}_{\text{emb }}^{\text{T}}{\;,\text{ target}}^{\text{T}})) \end{array} \end{array}$$ (3)

### The position encoding and transformer modules

Typically, contrastive loss from the previous steps can effectively capture the relationship between patches and gene expressions, allowing for ST prediction through inference. However, we innovatively consider the possible unreliability present in the original gene expression data. Due to the high spatial resolution, cellular-level ST data often suffer from missing values and inaccuracies. To tackle this, we leverage Transformer modules equipped with multi-head self-attention mechanisms and positional encoding [28]. These models are adept at learning relationships between different spots within an image and encoding spatial information, making them ideal for addressing the spatial dependencies in ST data.

We implement a Transformer-based autoencoder that simultaneously denoises and imputes gene-expression profiles while preserving each slide’s intrinsic spatial structure. A dedicated graph attention-based positional encoder further equips the network to capture long-range tissue dependencies without losing the fine-grained neighborhood cues. Following the previous step of generating gene expression embeddings ${\text{X}}_{\text{emb }}\in R^{\text{N}\times {\text{ dim }}_{\text{proj }}}$, we incorporate the Transformer autoencoder in our training procedure as follows.

For positional embeddings, we adopt conditional positional encodings (CPE), originally proposed in CPVT Inspired by SpaFormer, we replace visual convolutions with a graph convolution, using a graph attention network (GAT) to generate position embeddings from the spatial adjacency graph [29,30]. These embeddings serve as the CPE, encoding spatial neighborhoods and achieving translation invariance, aligning with the ST context. Specifically, for the gene expression matrix, the GAT positional encoding implemented by the TransformerConv layer from PyTorch Geometric, and can be formulated as follows:


$$\begin{array}{c} \begin{array}{c} {\text{Pos}}_{emb_{i}}=\sigma \left(BN\left({\text{W}}_{\text{1}}{\text{X}}_{\text{em}{\text{b}}_{\text{i}}}+\sum_{j\in N(i)}\;\;\alpha_{i,j}{\text{W}}_{\text{2}}{\text{X}}_{\text{em}{\text{b}}_{\text{j}}}\right)\right) \end{array} \end{array}$$ (4)

where ${\text{W}}_{\text{1}}$ and ${\text{W}}_{\text{2}}$ are weight matrices applied to the node feature of the current spot *i* and its neighbor *j*. The attention coefficients $\alpha_{i,j}$ are computed via multi-head dot product attention. The final input embedding into the Transformer can be represented as:


$$\begin{array}{c} \begin{array}{c} Emb={\text{X}}_{\text{emb}}+{\text{Pos}}_{\text{emb}} \end{array} \end{array}$$ (5)

Within the transformer architecture, the multi-head attention mechanism is central. Given the input embedding Emb, we define the input vectors as Q $=\text{Emb}\cdot {\text{W}}_{\text{Q}},\text{K}=\text{emb}\cdot {\text{W}}_{\text{K}}$, and $\text{V}=\text{Emb}\cdot {\text{W}}_{\text{V}}$, calculating the attention individually for each head, then concatenating the results and performing a linear transformation to obtain the multi-head attention:


$$\begin{array}{c} \begin{array}{c} \text{MultiHead}(Q,K,V)=\text{ Concat }({\text{head}}_{\text{1}},\ldots \;\;\;\;,\text{ head }\;h)\text{W}\\ where\;{\text{ head }}_{\text{i}}=\text{Attention}(\text{Q},\text{K},\text{V})=\text{softmax}\left(\frac{\text{Q}{\text{K}}^{\text{T}}}{\sqrt{{\text{d}}_{\text{k}}}}\right)\text{V} \end{array} \end{array}$$ (6)

After L layers of such transformer blocks, we obtain the final embedding of gene expression as $\hat{\text{x}}\in R^{\text{n}\times {\text{ dim }}_{\text{proj }}}$.

### The generalized autoencoder

Since our previous encoders E has already implemented feature extraction from the original input x to obtain $\hat{\text{x}}=$ e(x) a decoder D and its associated loss function is required. We first implement an autoencoder where the decoder consists of linear layers that project the embedding $\hat{\text{x}}\;$back to the same dimension as x. The reconstructed output is denoted as ${\text{x}}_{\text{reconst }}=$ $\text{d}(\hat{\text{x}})$. The root mean square error (rmse) loss function for this autoencoder can be written as:


$$\begin{array}{c} \begin{array}{c} L_{RMSE}=\sqrt{∥{\text{X}}_{\text{reconst}\;}-\text{X}{∥}^{\text{2}}} \end{array} \end{array}$$ (7)

Simultaneously, we also explore ZINB-based autoencoders [26]. Previous studies have demonstrated that the discrete and over dispersed nature of ST data, characterized by a high frequency of zero values, can be effectively modeled using the ZINB distribution [31,32]. In the ZINB framework, the decoder consists of three independent fully connected layers that estimate the three parameters associated with the ZINB distribution: the dropout rate π, the dispersion θ and the mean μ. These parameters are defined as follows:


$$\begin{array}{c} \begin{array}{c} \text{Disp }\theta =\text{Exp}\left(w_{\theta }\hat{X}\right) \end{array} \end{array}$$ (8)

$$\begin{array}{c} \text{Mean }\mu =\text{diag}\left(S_{i}\right)\times \text{exp}\left(w_{\mu }\hat{X}\right) \end{array}$$

These three parameters are used to calculate the ZINB probability mass function, which can be defined as:


$$\begin{array}{c} \begin{array}{c} \text{NB}(\text{x}|\mu ,\theta )=\frac{\Gamma (\text{x}+\theta )}{\text{x}!\Gamma (\theta )}{\left(\frac{\theta }{\theta +\mu }\right)}^{\theta }{\left(\frac{\mu }{\theta +\mu }\right)}^{\text{x}}\\ \text{ZINB}(\text{x}|\pi ,\mu ,\theta )=\pi \delta_{\text{0}}(\text{x})+(\text{1}-\pi )\text{NB}(\text{x}) \end{array} \end{array}$$ (9)

Where $\delta_{\text{0}}$ denotes the indicator function at zero. To ensure numerical stability during training,we add $\varepsilon =\text{1}\;\times \;{\text{1}\text{0}}^{-\text{1}\text{0}}$ to all logarithmic and division operations in the likelihood computation. We define the ZINB loss function as the sum of the negative log-likelihood of the ZINB distribution, formulated as:


$$\begin{array}{c} \begin{array}{c} L_{\text{ZINB}}=-\text{log}(\text{ZINB}(\text{X}|\pi ,\mu ,\theta )) \end{array} \end{array}$$ (10)

Following the computations mentioned above, we calculate the weighted sum of the losses from each module as the final loss function:


$$\begin{array}{c} \begin{array}{c} \text{ Loss }=L_{\text{cont}}+\alpha L_{\text{RMSE}}+\beta L_{\text{ZINB}} \end{array} \end{array}$$ (11)

where α = 0.1 and β = 0.25 denote the weight parameters of RMSE and ZINB loss, respectively.

With the trained model and fine-tuned UNI encoder, model inference and ST prediction can take place for each spot on the histology images. The inference procedure begins by dividing the histology image into N small patches, following the same preprocessing steps during training. These patches are then passed through the fine-tuned UNI image encoder and projected into the embedding space.

Simultaneously, the gene expressions and embeddings of all training spots/patches, precomputed during the training phase, serve as a reference [18]. As such, the reference bank will only include patches/ spots from the training set, and completely excludes spots/ patches from the held out test sets. For each test patch, we calculate its proximity to the reference spots using the Euclidean distance in the joint embedding space. We select k = 100 nearest reference spots and denote the final $X_{\text{pred }}$ as a weighted linear combination of the expression embeddings of these reference spots. The transformer autoencoder and ZINB reconstruction heads act as training-time regularizers that shape the learned joint embeddings space and are not used at inference. At prediction time, expression for each query patch is obtained solely through k-nearest-neighbor interpolation over the reference bank, without invoking the autoencoder or ZINB decoder.

### Hyperparameter settings

FOCST was trained with an initial learning rate of 0.001 and further adjusted with a learning rate scheduler and optimized with the Adam optimizer. We use a step size of 30, decay rate of 0.001 and dropout of 0.1. Loss function weights were set to α = 0.1 and β = 0.25 for RMSE and ZINB loss based on preliminary validation experiments (S6 Table). Temperature and KN values are determined through sensitivity analysis for both parameters, with k = 8 and τ = 1 chosen as final parameter values (S4 and S5 Tables). The UNI vision encoder was trainable (not Frozen) and updated via backpropagation alongside the expression encoder, project heads and decoders. For comparisons, all baselines were trained with their default parameters until convergence. Detailed hyperparameter list can be found in S1 Table.

FOCST requires substantial computational resources due to the large UNI foundation model and end-to-end fine-tuning strategy. FOCST was trained for 200 epochs using 3 A100 GPUs, with 80 GB of memory each. We adopted a distributed processing framework with 170 patches on each GPU for an effective batch size of 510. Each training run with 200 epochs took approximately 3 – 4 hours, with inference time being only around 5 minutes with a single A100 GPU.

### Performance evaluation of predicted ST profiles

We measure the accuracy of the predicted gene expression through PCC and SC.


$$\begin{array}{c} \begin{array}{c} \text{PCC}=\frac{\text{Cov}\left(X_{\text{pred }},\text{X}\right)}{\sqrt{\text{Var}\left(X_{\text{pred }}\right)\times \text{Var}(\text{X})}} \end{array} \end{array}$$ (12)

$$\begin{array}{c} \begin{array}{c} \text{SC}=\frac{\text{Cov}\left({rank(X}_{\text{pred }}),\text{rank}(\text{X})\right)}{\sqrt{\text{Var}\left(rank\left(X_{\text{pred }}\right)\right)\times \text{Var}(\text{rank}(\text{X}))}} \end{array} \end{array}$$ (13)

where Cov() is the covariance, and Var() is the variance. ${\text{X}}_{\text{pred}}\;$and X are the predicted and observed gene expression, respectively. To benchmark FOCST’s performance, we compared it against four established baselines: BLEEP, Hist2ST, HisToGene, and ST-Net. Hist2ST builds upon HisToGene and ST-Net and utilizes the same breast cancer dataset for training and validation as our study. Notably, our model incorporates key elements from these baselines. Specifically, we adopted the combination of ZINB and RMSE losses from Hist2ST and the reference-based inference approach for gene expression prediction from BLEEP [18,26].

FOCST generalization was assessed via leave-one-patient-out cross-validation (LOOCV) on the HER2 + dataset (N = 8 patients) and cSCC dataset (N = 4 patients). In each fold, the model was trained on all patients except one and evaluated on the held-out patient. We report the mean and 95% confidence interval across patients, calculated using the t-distribution.

For baseline comparisons, all methods were evaluated on held-out test sets not seen during training: HER2+ (2 patients, 6 images, 3,097 patches), cSCC (1 patient, 3 images, 1,691 patches), and Lung (1 image, 2017 patches)(S8 Table). To avoid pseudo replication arising from spatial autocorrelation among neighboring patches, predictions were aggregated to the image level prior to statistical testing, yielding N = 6, N = 3, and N = 1 independent observations, respectively. For the HER2 + dataset (N = 6 images), FOCST was compared against each of five baselines (BLEEP, Hist2ST, THItoGene, HisToGene, STNet) using one-sided paired Wilcoxon signed-rank tests (testing FOCST > baseline). To control for multiple comparisons across 30 tests (5 baselines × 3 evaluation levels × 2 correlation metrics), p-values were adjusted using the Benjamini-Hochberg false discovery rate (FDR) procedure at α = 0.05. For cSCC (N = 3) and Lung (N = 1), formal hypothesis testing was not performed because the minimum achievable p-values (0.125 and 1.0, respectively) exceed the significance threshold regardless of effect size. Results for these datasets are reported descriptively as evidence of generalizability.

### Patient treatment response prediction using imputed ST data

In order to assess the clinical relevance and FOCST’S ability to retain and impute biological relevant information in its predictions, the predicted ST data is subject to patient treatment response prediction. The Yale Trastuzumab response cohort from the Cancer imaging Archive will be used. The Her2 + patients were identified by retrospective search in the Yale Pathology electronic database, and patients’ HER2 + status was determined using the immunohistochemistry score of 3 + , as defined by The American Society of Clinical Oncology/College of American pathologists (ASCO/CAP) clinical practice guidelines. The database consists of a total of 192 patients with 99 HER2- and 93 HER2 + patients. Out of the 93 HER2 + patients, 85 patients are amongst a trastuzumab response cohort, which we use as our target dataset for evaluating the ability of FOCST generated ST data to generate treatment response predictions [33]. The 85 HER2 + invasive breast carcinoma patients received neoadjuvant targeted therapy with trastuzumab + /- pertuzumab prior to surgery. The database contains H&E stained histology images for each patient in addition to whether they are a responder (n = 36) or a non-responder (n = 49).

Due to heterogenous staining procedures, a stain normalization procedure was applied to ensure that histology images were standardized prior to processing. The patient O10-12717 was randomly selected as the reference for stain normalization. The luminosity of the reference image was first standardized using the LuminosityStandardizer function. Subsequently, a StainNormalizer using the Vahadane method was fitted to this reference image. The Vahadane technique involves decomposing the target image into two components using non-negative matrix factorization. A stain concentration matrix and a stain colour matrix are identified, enabling the function to adjust the image’s staining profile to match that of the reference image.

For each individual image, a grayscale mask was generated as part of the preprocessing pipeline to allow for better segmentation of image patches (**S2 Fig**). Initially, a Gaussian blur was applied to reduce noise and smooth the image. Following this, Otsu thresholding was used to automatically determine an optimal threshold value between 0 and 255. The Otsu thresholding algorithm works by minimizing the intra-class variance in the pixel intensity distribution, which effectively separates the foreground (tissue) from the background (**S2 Fig**). A patch generation algorithm was then applied to each image to generate patches of 224 x 224 pixels. Each image is divided into non-overlapping patches by iterating over its dimensions in steps equal to the patch size. Patches that extend beyond the image boundaries are discarded to ensure only complete patches are processed. For each patch, a tissue coverage is calculated as the ratio of tissue pixels (black regions on the image mask with a pixel value of 0) to the total number of pixels in the patch. Patches that meet a tissue-coverage threshold of 75% or greater are retained, and their pixel coordinates are recorded for subsequent ST imputation (S8 Table).

In order to generate single cell level ST profiles, an external framework called SpatialScope was used in conjunction with a reference scRNA dataset. The chosen reference dataset is from a single HER2-positive female breast cancer patient. The scRNA expression profile was sequenced using the Illumina NextSeq 500 platform, and comprises 2353 cells and 29,733 genes [33]. Using the FOCST predicted ST data, SpatialScope generates single-cell level ST data consisting of 29,733 genes. The expression profiles undergo preprocessing prior to highly variable gene (HVG) and marker gene identification. Genes expressed in fewer than 3 cells and cells expressing fewer than 100 genes are removed. The retained expression profiles are then normalized to 10,000 counts per cell, log-transformed, and scaled using functions from the Scanpy package.

To avoid feature selection leakage and obtain unbiased performance estimates, gene selection was performed independently within each cross-validation training fold with random seed set to 42**.** For each patient, marker genes for each of the seven cell types (PVL, CAFs, Endothelial, Myeloid, Plasmablasts, T-cells, and B-cells) were identified using the sc.tl.rank_genes_groups function with the Wilcoxon method, and HVGs were identified per cell type using the sc.pp.highly_variable_genes function with the flavor parameter set to ‘seurat’. Within each outer cross-validation fold, genes were aggregated across training patients by frequency, where the top 5 and top 10 most frequently appearing genes per cell type across training patients were selected as the final gene set. This approach ensures that gene selection is informed only by training data and produces robust, generalizable gene panels. Gene lists for each cell type were also combined to evaluate the predictive power of all cell types together.

Using the selected gene lists, pseudo-bulk expression features (mean expression across all cells per patient) were extracted and used as input for a treatment response classifier. A logistic regression model from the Scikit-Learn package was employed, and optimal hyperparameters (regularization type: L1, L2, or ElasticNet; regularization strength C ∈ {0.001, 0.01, 0.1, 1, 10}) were determined via a nested cross-validation design. The outer loop consisted of a repeated stratified 5-fold cross-validation with 10 repeats for a total of 50 outer test folds. For each outer split, gene selection was performed using only training patients, and a stratified 3-fold inner cross-validation determined the optimal hyperparameters. Performance was summarized using the ROC-AUC score on the outer test sets. The mean and 95% confidence interval were estimated using a two-sided Student’s t-interval across the 50 outer folds.

A baseline model based on histology images was developed to benchmark the gene expression-based classifier. In this approach, the UNI foundation model was employed as the image encoding backbone, paired with a multi-layer perceptron (MLP) for treatment response classification. For each patient, the corresponding stain-normalized histology image is resized to 224 × 224 pixels. UNI generates a 1024-dimensional feature embedding, which serves as input to the MLP. The classifier consists of a fully connected layer that projects the 1024-dimensional embedding to a 128-dimensional hidden space, followed by a ReLU activation function, a dropout layer (rate = 0.5), and a second fully connected layer that reduces the hidden dimension to a single output node. A sigmoid activation function produces the predicted probability of treatment response.

During training, the last transformer block and final layer normalization of UNI were fine-tuned while earlier layers remained frozen, allowing domain adaptation while preserving pretrained representations. The model was trained for 50 epochs using the Adam optimizer with a learning rate of 1 × 10^−4^ and binary cross-entropy loss. Model performance was evaluated using ROC-AUC. The image-based model was cross-validated using a stratified 5-fold cross-validation with 10 repeats, and 95% confidence intervals were constructed using a two-sided Student’s t-interval across the 50 folds.

## Results

### ST prediction

Performance evaluation was measured with Pearson correlation coefficient (PCC) values and Spearman’s rank Correlation (SC) values at three different levels: the top 50 highest correlated genes, all 785/171/415 genes for the respective dataset, and all patches constructed from a test image.

**Fig 2:**
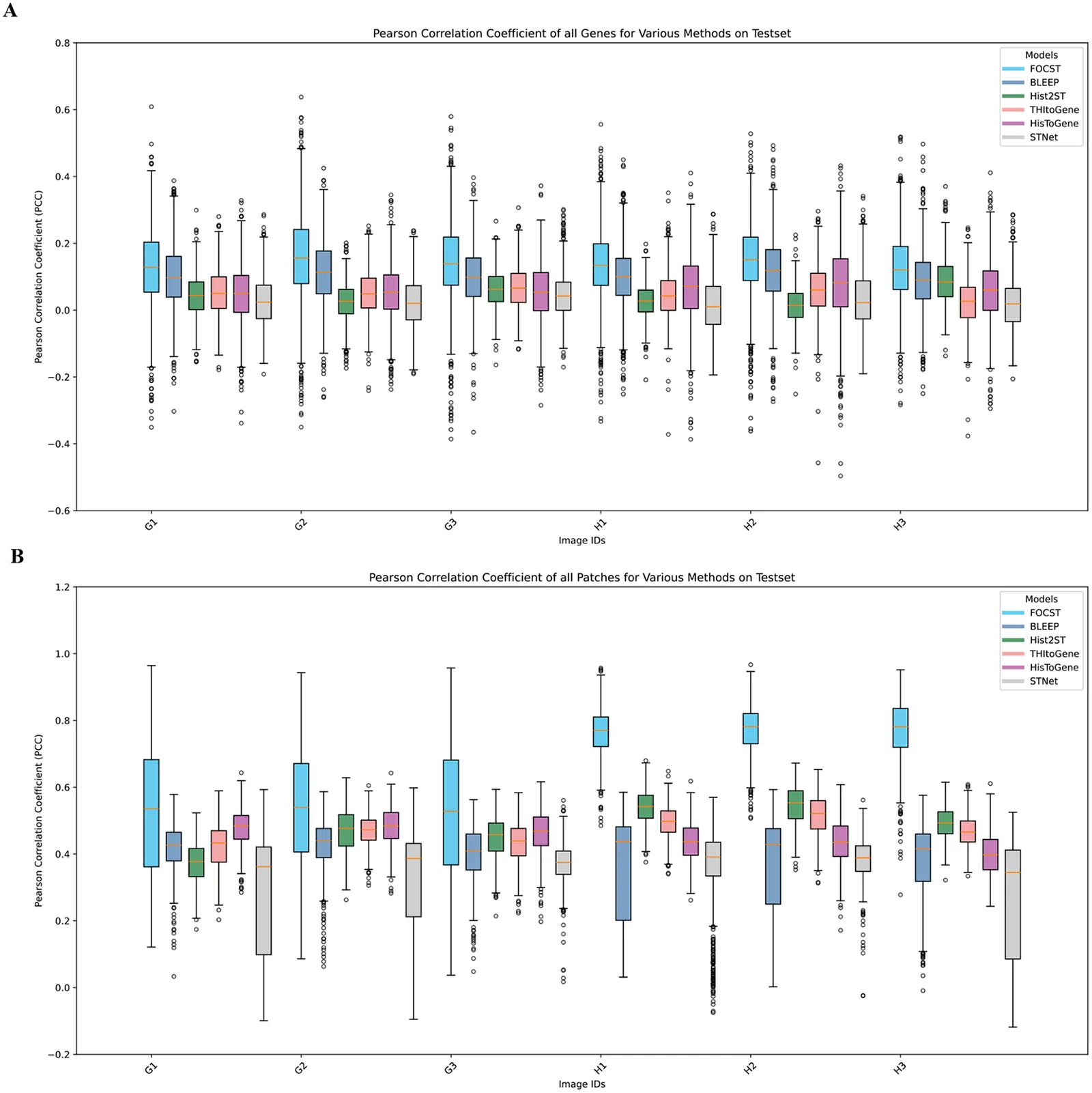
Pearson Correlation Coefficient (PCC) values for FOCST and baseline models evaluated on the HER2-positive test set. **A)** PCC values for all 785 genes in the dataset. **B)** PCC values for all generated patches from a test image.

Across the HER2-positive, cSCC, and Lung datasets, FOCST achieves consistently higher PCC and SC values than the other baseline models, with the largest gains often visible at the patch leve**l** (Table 2 and Fig 2). For instance, in the HER2-positive dataset, FOCST’s average patch‐level PCC is 0.648 ± 0.014, approximately 0.20 higher than the second‐best method, HisToGene at 0.449 ± 0.029. This can also be observed at the patch level in the cSCC dataset, where FOCST (0.529 ± 0.083) outperforms the second‐best method, THItoGene (0.502 ± 0.081), by about 0.027. In the Lung dataset (single test image), FOCST achieved a patch-level PCC of 0.388, outperforming the highest baseline THItoGene (0.257) by 0.131. Gene‐level correlations depict similar improvements: for HER2-positive, FOCST’s PCC is 0.139 ± 0.011, roughly 0.037 above the next‐highest model (BLEEP), while in cSCC, FOCST exceeds Hist2ST by around 0.01 in gene‐level PCC. For the Lung dataset, FOCST’s gene-level PCC of 0.093 exceeds THItoGene by 0.018. On the HER2-positive dataset, FOCST significantly outperformed all five baselines across all metrics (paired Wilcoxon signed-rank tests with Benjamini-Hochberg FDR correction across 30 comparisons, all adjusted p < 0.05, N = 6 images). For cSCC (N = 3 images) and Lung (N = 1 image), formal statistical testing was not performed due to limited sample sizes. However, FOCST’s consistent superior performance across these external datasets provides descriptive evidence for generalizability to alternate cancer types and histology images generated using different ST technologies.

**Table 2:** Method comparison across three datasets. Values represent mean ± SD of PCC and SC aggregated to the image level. HER2+ (N = 6 images): *indicates FDR-adjusted p < 0.05 (paired Wilcoxon signed-rank test, Benjamini-Hochberg correction across 30 comparisons). cSCC (N = 3 images) and Lung (N = 1 image): statistical testing not performed due to limited sample sizes; results reported descriptively. Bold indicates highest performance per dataset.

Dataset	Method	Patch PCC	Patch SC	Gene PCC	Gene SC	Top 50 PCC	Top 50 SC
HER2+	FOCST	0.648 ± 0.014*	0.593 ± 0.092*	0.139 ± 0.011*	0.139 ± 0.012*	0.388 ± 0.030*	0.380 ± 0.034*
(n = 6)	BLEEP	0.391 ± 0.022	0.285 ± 0.023	0.102 ± 0.009	0.102 ± 0.008	0.299 ± 0.017	0.291 ± 0.013
	Hist2ST	0.425 ± 0.066	0.362 ± 0.031	0.067 ± 0.028	0.061 ± 0.028	0.200 ± 0.059	0.188 ± 0.048
	THItoGene	0.468 ± 0.090	0.352 ± 0.047	0.050 ± 0.010	0.053 ± 0.009	0.192 ± 0.018	0.185 ± 0.014
	HisToGene	0.449 ± 0.029	0.335 ± 0.019	0.059 ± 0.010	0.064 ± 0.009	0.243 ± 0.026	0.241 ± 0.025
	STNet	0.329 ± 0.046	0.249 ± 0.027	0.027 ± 0.010	0.035 ± 0.019	0.158 ± 0.034	0.204 ± 0.029
cSCC	FOCST	**0.529 ± 0.083**	**0.471 ± 0.035**	**0.182 ± 0.032**	**0.184 ± 0.019**	**0.294 ± 0.046**	**0.292 ± 0.026**
(n = 3)	BLEEP	0.332 ± 0.031	0.278 ± 0.022	0.078 ± 0.013	0.087 ± 0.012	0.179 ± 0.027	0.188 ± 0.022
	Hist2ST	0.491 ± 0.041	0.350 ± 0.035	0.172 ± 0.026	0.175 ± 0.013	0.285 ± 0.040	0.287 ± 0.022
	THItoGene	0.502 ± 0.081	0.441 ± 0.068	0.174 ± 0.080	0.163 ± 0.076	0.289 ± 0.083	0.279 ± 0.078
	HisToGene	0.380 ± 0.032	0.295 ± 0.022	-0.006 ± 0.003	-0.008 ± 0.004	0.068 ± 0.001	0.074 ± 0.004
	STNet	0.423 ± 0.047	0.326 ± 0.040	0.081 ± 0.009	0.088 ± 0.011	0.164 ± 0.009	0.182 ± 0.008
Lung	FOCST	**0.388 ± 0.00**	**0.290 ± 0.00**	**0.093 ± 0.00**	**0.073 ± 0.00**	**0.232 ± 0.00**	**0.229 ± 0.00**
(n = 1)	BLEEP	0.202 ± 0.00	0.186 ± 0.00	0.043 ± 0.00	0.046 ± 0.00	0.174 ± 0.00	0.183 ± 0.00
	Hist2ST	0.033 ± 0.00	0.029 ± 0.00	0.036 ± 0.00	0.042 ± 0.00	0.129 ± 0.00	0.158 ± 0.00
	THItoGene	0.257 ± 0.00	0.285 ± 0.00	0.075 ± 0.00	0.080 ± 0.00	0.182 ± 0.00	0.193 ± 0.00
	HisToGene	0.022 ± 0.00	0.018 ± 0.00	0.013 ± 0.00	0.016 ± 0.00	0.063 ± 0.00	0.063 ± 0.00
	STNet	0.020 ± 0.00	0.019 ± 0.00	0.001 ± 0.00	0.001 ± 0.00	0.074 ± 0.00	0.078 ± 0.00

A distinct difference can be observed between the patch level PCC values for patient H and patient G. This may be due to the small sample size that was used during training, as the prediction results still have varying degrees of accuracy. Through training on a larger dataset, the model would learn to generalize better between different patients and generate more stable results, with less variability.

To assess generalization, FOCST was evaluated using leave-one-patient-out cross-validation (LOOCV) on two datasets: HER2 + breast cancer (N = 8 patients) and cSCC (N = 4 patients) (Tables 3 and S2 and S3 Table). On the HER2 + dataset, FOCST achieved robust patch-level performance with a mean PCC of 0.577 (95% CI: 0.471–0.683) and SC of 0.454 (95% CI: 0.369–0.538). Gene-level correlations showed a mean PCC of 0.161 (95% CI: 0.070–0.251) across all 785 genes, increasing to 0.371 (95% CI: 0.214–0.528) for the top 50 most predictable genes. Performance varied across patients (Patch PCC range: 0.455–0.787), likely reflecting differences in tissue heterogeneity and sample quality. On the cSCC dataset, FOCST demonstrated comparable generalization with a mean patch-level PCC of 0.465 (95% CI: 0.324–0.606). Notably, gene-level performance was higher on cSCC (PCC = 0.195, 95% CI: 0.115–0.275) compared to HER2+ (PCC = 0.161), despite the smaller gene panel (171 vs. 785 genes). This cross-cancer validation provides evidence that FOCST generalizes beyond the breast cancer domain to other cancer types and ST technologies.

**Table 3:** FOCST performance via leave-one-patient-out cross-validation. Values represent mean PCC and SC with 95% confidence intervals across patients (HER2 + : N = 8 patients, df = 7; cSCC: N = 4 patients, df = 3). Confidence intervals calculated using the t-distribution.

Dataset	N	Metric	Mean	95% CI
**HER2+**	8	Patch PCC	0.577	(0.471, 0.683)
		Gene PCC	0.161	(0.070, 0.251)
		Top 50 PCC	0.371	(0.214, 0.528)
		Patch SC	0.454	(0.369, 0.538)
		Gene SC	0.142	(0.051, 0.234)
		Top 50 SC	0.368	(0.216, 0.519)
**cSCC**	4	Patch PCC	0.465	(0.324, 0.606)
		Gene PCC	0.195	(0.115, 0.275)
		Top 50 PCC	0.295	(0.113, 0.477)
		Patch SC	0.420	(0.329, 0.511)
		Gene SC	0.201	(0.077, 0.325)
		Top 50 SC	0.339	(0.127, 0.551)

To validate that FOCST predictions capture meaningful spatial structure, we computed Moran’s I, a measure of spatial autocorrelation, for each gene across test images. Positive Moran’s I values indicate that neighboring spots exhibit similar expression levels, reflecting spatially coherent predictions rather than random noise. FOCST achieved an overall Moran’s I of 0.528 ± 0.102 with nearly 100% of genes showing significant spatial autocorrelation (p < 0.05) across all test images (Table 4). These results confirm that the graph attention-based positional encoding effectively captures local spatial dependencies in predicted expression profiles.

**Table 4:** Spatial autocorrelation of FOCST predictions on HER2-positive test set.

Image	Moran’s I	% Significant (p < 0.05)
G1	0.465 ± 0.111	100%
G2	0.420 ± 0.114	99.9%
G3	0.407 ± 0.0087	100%
H1	0.648 ± 0.084	100%
H2	0.655 ± 0.073	100%
H3	0.570 ± 0.073	100%

### Ablation studies

We conducted ablation studies to demonstrate the contribution of each proposed module in our architecture. We investigate the contributions of the histopathology foundation model, UNI and each individual loss component in our combined loss function.

We assess the impact of leveraging UNI and the contributions of our positional encoding transformer. Replacing UNI with a ResNet50 module leads to a modest decline in performance, particularly at the patch level (Table 5). This is expected, as patch-level predictions rely more heavily on local morphological features, where the choice of visual encoder is critical. However, performance degrades even further when using UNI in a zero-shot setting and using the knowledge for its strong pretraining on a massive histopathology corpus. This outcome underscores the necessity of domain-specific fine-tuning: without this step, UNI’s representations lack the BC specific adaptation required for accurate spatial transcriptomics prediction. Notably, the zero-shot UNI underperforms even relative to a fine-tuned ResNet50 a far simpler architecture, with patch level PCC values of 0.571 compared to ResNet50 with 0.615. In contrast, when the positional embedding was ablated, performance degraded across all metrics again but particularly for the mean PCC value for all genes and top 50 genes (Table 5). This suggests that the incorporation of our positional encoding transformer is crucial for accurately capturing spatial relationships necessary for the correlation between the predicted and ground truth expression. Despite this, the presence of UNI still proves a higher average patch PCC compared to the model with ResNet50, further demonstrating its capabilities as an image encoder for histopathology vision tasks.

**Table 5:** Ablation Study of Implemented Modules. Mean PCC values are calculated for the HER2-positive dataset for the patch level, gene level, and the top 50 highest correlated genes. Bolded represents the lowest performing ablation experiment.

Method	Patch	Gene	Top 50
FOCST	0.648	0.139	0.388
UNI Zero-shot	**0.571**	**0.111**	**0.304**
w/o UNI (ResNet50)	0.615	0.112	0.378
w/o positional embedding	**0.586**	**0.052**	**0.197**

We also evaluated the contribution of each individual loss component, including root mean squared error (RMSE) loss and Zero-inflated negative binomial (ZINB) loss. The combined loss achieved the highest average PCC values across all levels, while omitting the ZINB loss resulted in the lowest average PCC (Table 6). Although there is a decrease in performance with the absence of RMSE loss, the magnitude is not as great. These results provide evidence for the use of a combined loss function to improve the accuracy of ST predictions.

**Table 6:** Ablation Study of Loss Components. Mean PCC values are calculated for the HER2-positive dataset for the patch level, gene level, and the top 50 highest correlated genes. Bolded represents the lowest performing ablation experiment.

Method	Patch	Gene	Top 50
FOCST	0.648	0.139	0.388
w/o RMSE Loss	0.644	0.021	0.209
w/o ZINB Loss	**0.565**	**0.001**	**0.166**

### Cancer gene visualization

Visualizations for two key cancer genes FASN and GNAS are provided. The GNAS gene encodes for a G-protein alpha subunit involved in signal transduction. In the context of BC, it plays a key role in cell proliferation and hormone signaling. Dysregulation and altered expression of this gene has been seen to contribute to tumor growth and endocrine resistance. FASN represents fatty acid synthase, a key enzyme that is responsible for de novo lipid biosynthesis. It is often overexpressed in aggressive BC and is associated with tumor progression, metabolic reprogramming, and poor prognosis.

**Fig 3:**
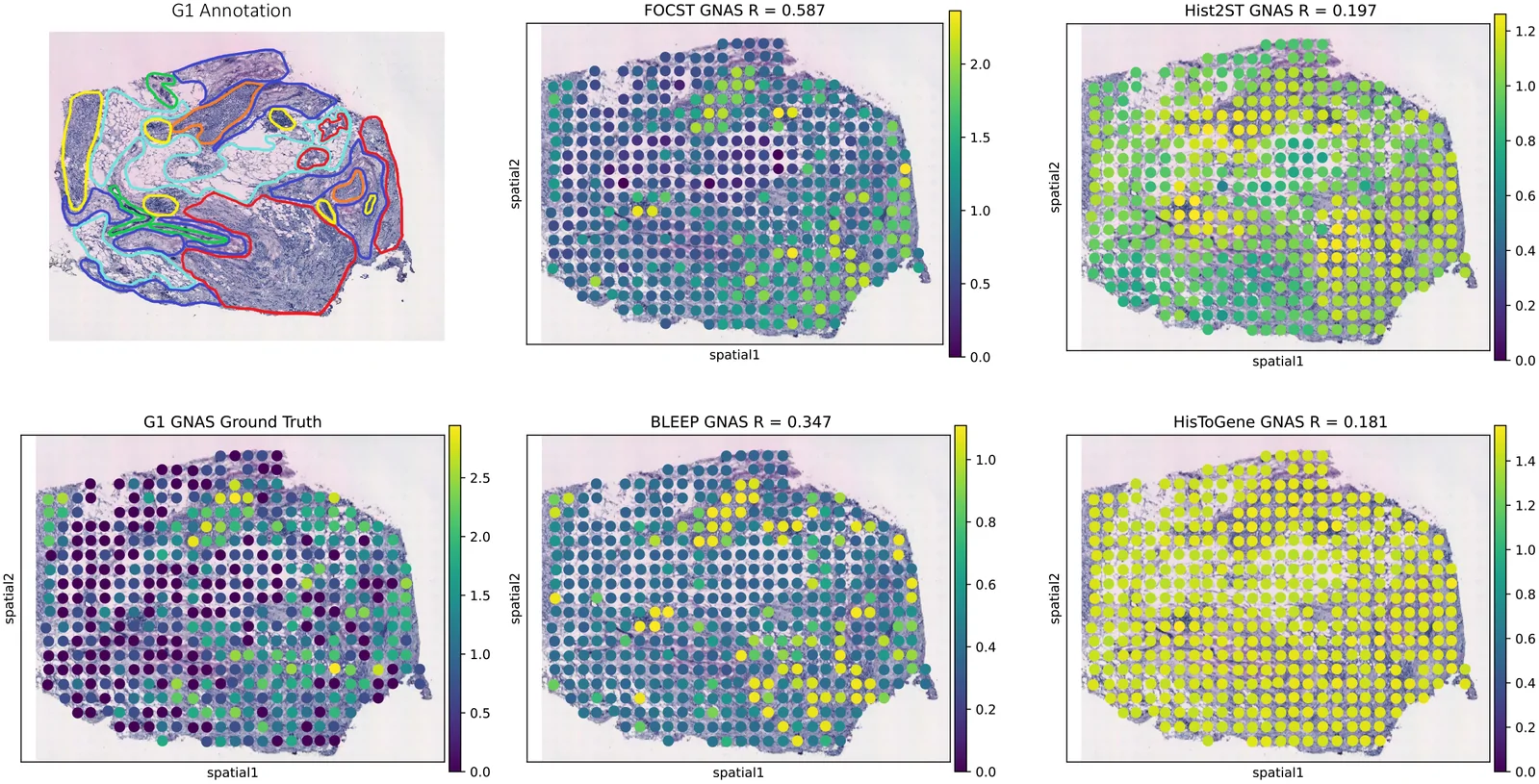
Visualization of GNAS expression profile. Comparison between ground truth expression levels, pathologist-annotated regions, FOCST, and baseline models.

**Fig 4:**
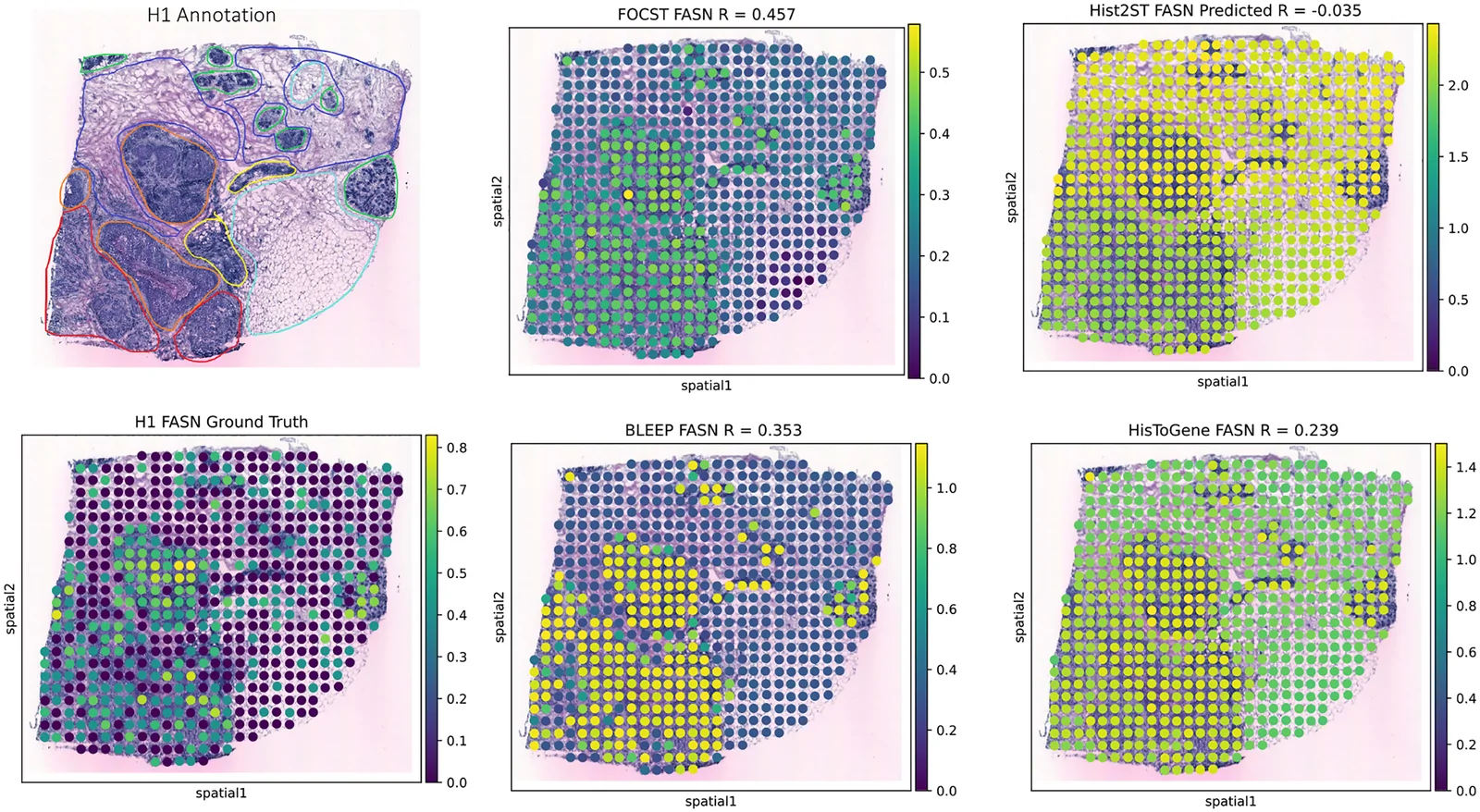
Visualization of FASN expression profile. Comparison between ground truth expression levels, pathologist-annotated regions, FOCST, and baseline models.

In both visualizations, the spatial gene expression predictions for FASN and GNAS are shown alongside the ground truth expression profiles and pathologist-annotated regions. In the ground truth panels, both genes exhibit well-defined and spatially localized expression patterns, likely corresponding to metabolically active or proliferative tumor subregions. Among the baseline models, HisToGene fails to capture these structured patterns, producing noisy, spatially diffuse predictions with limited morphological alignment. Hist2ST offers modest improvements by incorporating local spatial modeling, but its outputs remain overly smooth and lack the spatial granularity seen in the true expression maps. BLEEP correctly identifies broader regions of increased expression; however, it struggles to distinguish fine-grained variations within those regions, resulting in a loss of expression-level differences across individual patches. In contrast, FOCST demonstrates the highest degree of visual congruence with the ground truth for both genes. Not only does FOCST accurately capture the boundaries and location of regions with elevated expression, but it also successfully reconstructs intra-region variability, reflecting subtle expression differences between neighboring image patches (Figs 3 and 4). In addition, when comparing to the annotated images for both genes, marked regions of interest have increased expression levels while unmarked regions demonstrate lower to no expression levels. More importantly, FOCST’s predictions preserve the spatial specificity observed in pathology for both FASN and GNAS, where expression boundaries closely align with annotated tissue structures. Baseline methods only demonstrate noisy predictions that do not mark these tissue boundaries as clearly as FOCST. These observations are consistent with previous PCC and SC measures of the accuracy of ST predictions, as FOCST demonstrates the highest correlation at the patch level.

### Treatment response prediction with imputed ST profiles

**Fig 5:**
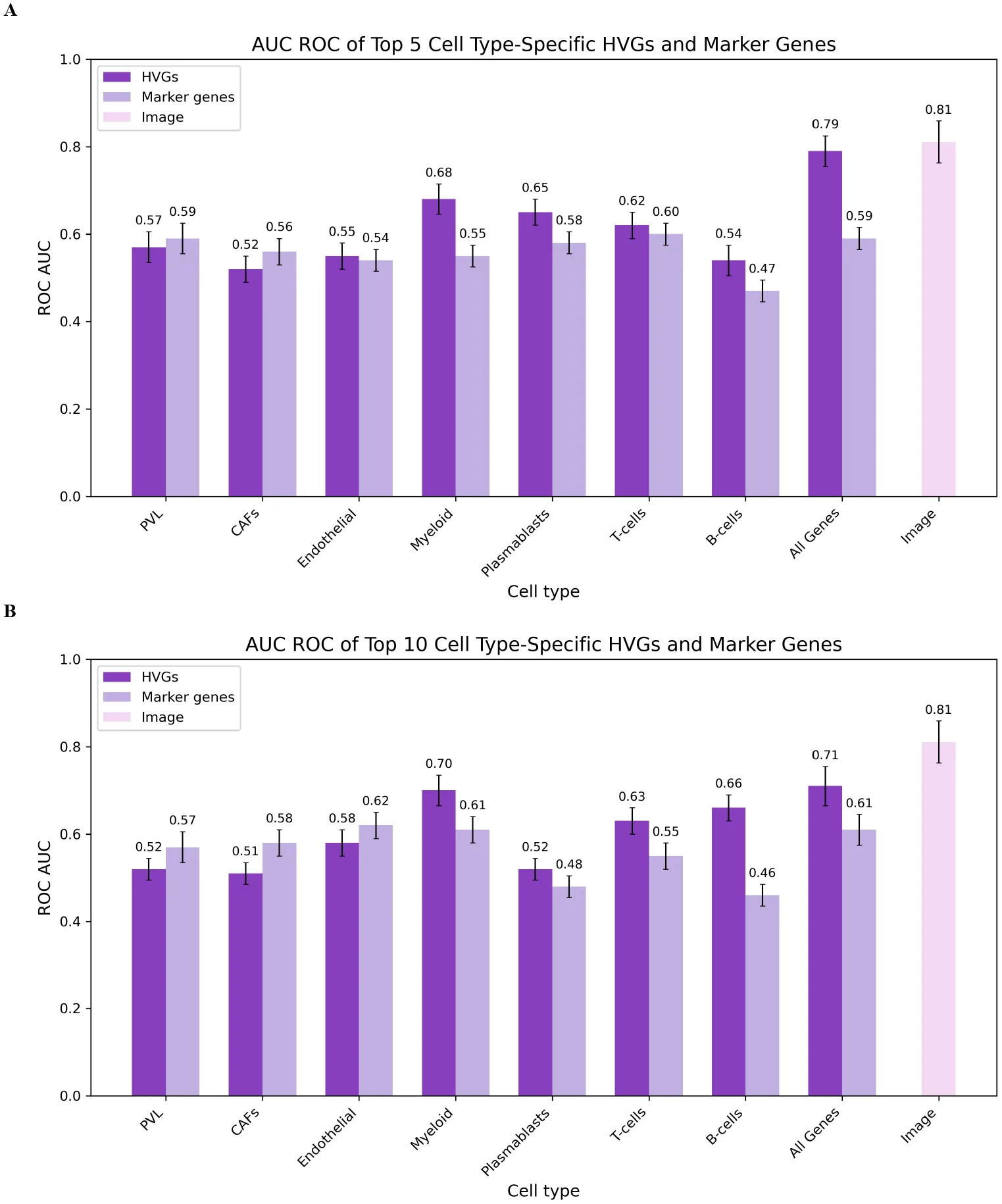
Trastuzumab Treatment Response Prediction Results. A) ROC-AUC of the top 5 marker and highly variable genes benchmarked against classifier using UNI generated embeddings. **B)** ROC-AUC of the top 10 marker and highly variable genes. Reported are the mean and 95% confidence intervals constructed through a two-sided student’s t-interval.

To assess the clinical utility of our predicted ST profiles, we evaluated treatment response prediction performance across cell types using gene sets derived from the FOCST-inferred expression matrix. For each major cell type, we constructed binary classifiers to distinguish responders from non-responders using either the top cell type-specific marker genes or HVGs derived from the single cell ST data that was obtained using an external deconvolution tool SpatialScope [34], and FOCST generated spot level ST data (S1 Fig). Performance was quantified using the area under the receiver operating characteristic curve (ROC-AUC), and results are shown for both the top 5 and top 10 genes per cell type to evaluate sensitivity to gene set size (Fig 5).

In the top 5 gene setting, HVGs generally outperformed marker genes across most cell types. Notably, myeloid and plasmablast HVG sets achieved the highest individual cell-type AUCs of 0.68 (95% CI: 0.645–0.715) and 0.65 (95% CI: 0.620–0.680), respectively, indicating strong discriminative power for treatment response (Fig 5A). T-cell HVGs also performed well with an AUC of 0.62 (95% CI: 0.590–0.650). In contrast, marker genes showed more modest performance, with T-cells and PVL achieving the highest marker-based AUCs of 0.60 and 0.59, respectively. Combining HVGs from all cell types yielded the strongest classification performance, achieving an AUC of 0.79 (95% CI: 0.755–0.825), substantially outperforming the combined marker gene set (AUC = 0.59, 95% CI: 0.565–0.615).

Expanding to the top 10 genes per cell type served as a sensitivity analysis to evaluate whether broader gene sets improve classification performance (Fig 5B). Myeloid HVGs remained the top-performing individual cell type, with AUC increasing to 0.70 (95% CI: 0.665–0.735). Notable improvements were observed for B-cell HVGs (AUC increased from 0.54 to 0.66) and T-cell HVGs (0.62 to 0.63). However, plasmablast HVGs showed decreased performance (0.65 to 0.52), suggesting that the additional genes introduced noise rather than signal for this cell type. For marker genes, endothelial cells showed improved performance at the top 10 setting (AUC = 0.62, 95% CI: 0.590–0.650). The combined HVG classifier achieved an AUC of 0.71 (95% CI: 0.685–0.755) at the top 10 setting, while combined markers achieved 0.61 (95% CI: 0.575–0.645).

The baseline model used histology images as input, with a fine-tuned UNI foundation model backbone. This image-based classifier achieved an AUC of 0.81 (95% CI: 0.763–0.858), comparable to the combined top 5 HVG classifier (AUC = 0.79). This indicates that the FOCST-derived single-cell ST profiles capture clinically relevant information approaching the predictive power of direct morphological features, while additionally enabling cell type-specific interrogation not possible with image-based approaches alone.

## Discussion

### ST predictions

FOCST is a novel framework for predicting ST profiles directly from readily available histology images. It includes a state-of-the-art histopathology foundation model for visual feature extraction, a graph neural network (GNN) for expression encoding, and a graph-attention–based positional encoder that captures both local and global spatial relationships. Together, these encoders underpin FOCST’s contrastive-learning strategy and enable highly accurate ST imputation. We benchmarked FOCST against multiple baselines on HER2-positive and cSCC datasets, evaluating performance with PCC and SC values. FOCST attained the highest patch-level PCC and SC scores on both datasets. Ablation studies confirmed that each architectural component, including image encoder, positional encoder, and contrastive objective, contributes meaningfully to the overall gain (S4–S7 Tables). To assess clinical utility, we subjected the imputed ST data to downstream analyses. SpatialScope generated single-cell–resolution profiles, from which we compiled cell-type-specific lists of HVG and marker genes. Using the combined HVG list across all cell types as features, a binary classifier predicting trastuzumab response achieved a ROC-AUC of 0.79. These results highlight both the robustness of FOCST’s predictions and their potential to inform treatment decisions.

While FOCST shows promising results, one key limitation lies in its reliance on spot-level ST data, where each sequencing spot encompasses 10–12 cells. Downstream applications, such as cell type-specific analysis or spatial deconvolution typically require single-cell resolution, which introduces an additional computational burden. Currently, we address this by applying SpatialScope, a post hoc method that decomposes spot-level predictions into pseudo-single-cell profiles. However, one major limitation of this approach is that any inaccuracies in FOCST’s patch-level predictions can negatively impact SpatialScope’s downstream reconstruction. Additionally, SpatialScope’s performance is highly dependent on the reference scRNA-seq dataset used and its ability to model expression distributions across cell types. This is evident in the clustering results where cells generated by SpatialScope appear skewed away from the centroids of their corresponding reference cell type clusters (S1 Fig). Such deviations suggest a mismatch between the inferred and true expression profiles, which likely impacted downstream performance. In particular, this discrepancy may have been a significant limiting factor for the treatment response classifier, as accurate gene expression levels are not only essential for constructing reliable gene sets but also serve as critical input features for classification. A related limitation concerns the reference bank used at inference. Because FOCST predicts expression by interpolating over the k = 100 nearest training spots in the joint embedding space, the size and composition of this bank may influence prediction quality. Future subsampling analyses that vary reference bank size and composition would characterize this dependence.

A major future direction is the development of an end-to-end architecture that directly infers single-cell–level ST from histology images, eliminating the need for separate deconvolution tools [35]. A further constraint stems from the reference-based inference strategy as predictions are formed by interpolating over expression vectors stored in the reference bank. FOCST can only predict genes present in the training ST panel and is unable to perform zero-shot prediction for unseen genes. Revisiting gene expression foundation models to predict beyond the observed gene panel could serve as a future direction. This could significantly reduce error propagation, increase accuracy of predicted ST profiles, and align better with the resolution required for downstream clinical analyses. Another future direction lies in the exploration of emerging foundation models, such as UNI-2, and Prov-GigaPath, which may offer stronger and more transferable visual representations. Evaluating FOCST’s performance across different vision backbones could further refine its prediction accuracy and adaptability.

A further limitation concerns the statistical evaluation of spatial performance. We computed Moran’s I per gene to confirm that FOCST’s predictions are spatially coherent rather than spatially random; however, this statistic characterizes the spatial structure of the predictions themselves and does not test whether FOCST outperforms competing methods under a spatially aware null. Because expression at neighboring spots is autocorrelated, a fully rigorous comparison would evaluate model superiority against a spatially constrained permutation null, in which predicted expression is permuted across locations in a way that preserves local spatial dependencies rather than treating spots as independent. Incorporating such a test represents a valuable direction for more rigorous future benchmarking. Moreover, expanding our validation to new large-scale spatial datasets such as HEST1K (comprising over 1,200 spatial transcriptomics profiles) will provide deeper insights into model generalization across tissue types and imaging modalities.

### Treatment response prediction

The superior performance of HVGs over marker genes suggests that intra-cell type variability may be more informative for predicting treatment response than inter-cell type specificity. In the provided analysis, HVGs were identified using scanpy.pp.highly_variable_genes, which selects genes based on their expression dispersion within a cell type. This method prioritizes genes that show high variability across cells of the same type, which captures the heterogeneity that may reflect underlying biological processes such as metabolic activity, stress response, or pathway activation, all of which may influence an individual’s susceptibility to trastuzumab treatment.

In contrast, marker genes were obtained using scanpy.tl.rank_genes_groups, which identifies genes that are differentially expressed between cell types using Wilcoxon rank-sum. This method is designed to highlight genes that are highly expressed in one cell type relative to others, making them useful for distinguishing cell identities. However, because the selection is based on inter-cell type differences rather than intra-cell type variability, the resulting gene list may not reflect expression patterns that are relevant to treatment response. As such, the expression levels of these marker genes may fail to capture the heterogeneity or functional state within a given cell type, which could be more predictive of therapeutic outcome.

When examining genes within each cell type, HVGs for myeloid cells, plasmablasts, and T-cells yielded the highest AUCs (0.68, 0.65, 0.62) (Fig 5). These cell types likely rank highest because their expression profiles capture broad features of the anti-tumour immune response that relates to trastuzumab. Plasmablast signals reflect humoral activity, myeloid signatures summarize innate effector and regulatory states, and T cell profiles reflect activation and cytotoxic potential. Variation across these compartments may encode both baseline immune readiness and treatment related changes, providing richer predictive signals than other lineages. The prominence of myeloid, plasmablast, and T-cell signatures is consistent with the established role of the immune microenvironment in HER2-positive breast cancer, where innate effector, humoral, and adaptive cytotoxic compartments shape the anti-tumour response [4].

While generating inferred single-cell profiles and cell-type–specific gene sets require additional computational overhead, the resulting gains in interpretability and the ability to perform downstream, cell-resolved analyses provide a strong justification for this approach. Importantly, the performance gap between reduced gene sets and imaging-based models suggests that further improvements may be achievable as the accuracy of single-cell inference improves. SpatialScope was selected because prior reports describe higher accuracy than other methods [34]. While we did not perform an internal comparison here, future studies should benchmark alternative single-cell imputation approaches while accounting for computational cost and run time.

Future work should also examine the top ten gene lists with pathway and network analysis to explain why certain cell types perform better than others. Finally, although the reference normalization image was chosen at random, we do not expect major changes in classifier performance. The FOCST backbone is a foundation model trained on a large and diverse collection of histology images that span scanners, staining protocols, and acquisition settings. Changes to the reference slide mainly affect low level colour representation rather than the morphological features that drive predictions, so the extracted embeddings remain stable and discriminative.

### Computational costs and training times

FOCST’s training requires 3 × A100 GPUs (80GB each) for approximately 3–4 hours per 200-epoch run, compared to simpler baselines (ST-Net, HisToGene) trainable on single GPUs in 1 hour. However, THItoGene, another Vision Transformer-based method, showed similar training costs (~2 hours), indicating computational overhead stems from the transformer architecture rather than FOCST-specific components. This increased training cost is justified by substantial performance gains as FOCST achieves patch PCC of 0.648, a 44% improvement over the next-best method (HisToGene: 0.449). More importantly, training time and resource consumption is a one time cost. Once trained, FOCST enables rapid inference (~5 minutes per patient on a single A100 GPU) for unlimited new samples.

## Conclusion

We present FOCST, a foundation model-based framework that accurately predicts spatial transcriptomics profiles from routine histology images through three key innovations: leveraging UNI for superior visual feature extraction, employing GAT-derived positional encoding to capture tissue neighborhood structure, and optimizing a contrastive learning objective for robust cross-modal alignment. FOCST achieves state-of-the-art performance across three cancer types (HER2 + breast cancer, cutaneous squamous cell carcinoma, and lung adenocarcinoma), with predicted profiles enabling clinically relevant downstream analyses including treatment response prediction (ROC-AUC = 0.79). By combining the power of foundation models with spatially-aware learning, FOCST offers a cost-effective, scalable approach to generate spatial transcriptomics insights from widely available histology images, with broad implications for cancer research, precision medicine, and clinical translation.

## Supporting information

S1 Fig SpatialScope Clustering Results. A) Clustered cells from SpatialScope generated cells and all cells from reference dataset. B) Labeled clustering results based on cell types from reference dataset.
(DOCX)

S2 Fig Preprocessing of Yale trastuzumab histology slides. A) original unprocessed H&E stained image B) Black and white image mask C) Stain normalized histology image.
(DOCX)

S1 Table Hyperparameters of FOCST and all baseline models. (DOCX)

S2 Table LOOCV results for FOCST by patient_ID with HER2-positive dataset. PCC reported at the patch, gene, and top 50 gene level.
(DOCX)

S3 Table LOOCV results for FOCST by patient_ID with cSCC dataset. PCC reported at the patch, gene, and top50 gene level.
(DOCX)

S4 Table Sensitivity Study of Temperature setting. Mean PCC values are calculated for the HER2-positive dataset for the patch level, gene level, and the top 50 highest correlated genes. Bolded represents the best performing selection.
(DOCX)

S5 Table Sensitivity study of nearest neighbor values. Mean PCC values are calculated for the HER2-positive dataset for the patch level, gene level, and the top 50 highest correlated genes. Bolded represents the best performing selection.
(DOCX)

S6 Table Sensitivity study of α and β values. Mean PCC values are calculated for the HER2-positive dataset for the patch level, gene level, and the top 50 highest correlated genes. Bolded represents the best performing selection.
(DOCX)

S7 Table FOCST pipeline with Prov-Gigapath backbone as image encoder. Mean PCC values are calculated for the HER2-positive dataset for the patch level, gene level, and the top 50 highest correlated genes. Bolded represents highest scoring.
(DOCX)

S8 Table Preprocessing checklist. (DOCX)
