# Supplementary material for: Spatially guided translation from histology images to transcriptomic profiles using foundation model-driven contrastive learning
Source: PLoS Comput Biol. 2026 Sep 8;22(9):e1014762. doi: 10.1371/journal.pcbi.1014762 (PMC13568522; doi:10.1371/journal.pcbi.1014762)
Supplement: Supplementary file 1 [file pcbi.1014762.s001.docx]

**S1 Fig** . SpatialScope Clustering Results. A) Clustered cells from SpatialScope generated cells and all cells from reference dataset. B) Labeled clustering results based on cell types from reference dataset
