# Supplementary material for: Spatially guided translation from histology images to transcriptomic profiles using foundation model-driven contrastive learning
Source: PLoS Comput Biol. 2026 Sep 8;22(9):e1014762. doi: 10.1371/journal.pcbi.1014762 (PMC13568522; doi:10.1371/journal.pcbi.1014762)
Supplement: Supplementary file 2 [file pcbi.1014762.s002.docx]

**S2 Fig .** Preprocessing of Yale trastuzumab histology slides. A) original unprocessed H&E stained image B) Black and white image mask C) Stain normalized histology image.
