# Supplementary material for: Spatially guided translation from histology images to transcriptomic profiles using foundation model-driven contrastive learning
Source: PLoS Comput Biol. 2026 Sep 8;22(9):e1014762. doi: 10.1371/journal.pcbi.1014762 (PMC13568522; doi:10.1371/journal.pcbi.1014762)
Supplement: Supplementary file 3 [file pcbi.1014762.s003.docx]

| FOCST | lr 0.001;  weight_decay 0.001;  patience 5; step 30;  factor 0.2;  image_embedding_dim 1024;  spot_embedding_dim 785/171/415;  rmse_coef 0.1;  zinb_coef 0.1;  temperature 1.0;  image_size 224;  num_projection_layers 1;  projection_dim 256;  dropout 0.1;  num_neighbors 4;  num_epochs 200 |
| --- | --- |
| BLEEP | lr 0.001;  weight_decay 0.001;  patience 2;  step 30; factor 0.5;  image_embedding_dim 2048;  spot_embedding_dim 785/171;  temperature 1.0;  image_size 224;  num_projection_layers 1;  projection_dim 256;  dropout 0.1;  num_epochs 100 |
| Hist2ST | lr 0.00001;  channel 32;  depth 2,8,4;  num_attention_heads 16;  kernel_size 5;  image_size 224;  dropout 0.2;  num_epochs 350 |
| THItoGene | lr 0.00001;  route_dim 64;  caps 20;  heads 16, 8;  n_layers 4;  n_genes: 785, 171, 415;  num_epoch 300 |
| HisToGene | lr 0.0001;  channel 32;  depth 2,8,4;  num_attention_heads 16;  kernel_size 5;  image_size 224;  num_layers 4;  dropout 0.1;  num_epochs 200 |
| STNet | lr 0.0001;  hidden_dim 2048;  num_layers 8;  num_position 64;  dropout 0.1;  num_epochs 200 |

**S1 Table**. Hyperparameters of FOCST and all baseline models.
