# Supplementary material for: Spatially guided translation from histology images to transcriptomic profiles using foundation model-driven contrastive learning
Source: PLoS Comput Biol. 2026 Sep 8;22(9):e1014762. doi: 10.1371/journal.pcbi.1014762 (PMC13568522; doi:10.1371/journal.pcbi.1014762)
Supplement: Supplementary file 4 [file pcbi.1014762.s004.docx]

**S2 Table.** LOOCV results for FOCST by patient_ID with HER2-positive dataset. PCC reported at the patch, gene, and top 50 gene level.

|  | **A** | **B** | **C** | **D** | **E** | **F** | **G** | **H** |
| --- | --- | --- | --- | --- | --- | --- | --- | --- |
| **Patch PCC** | 0.455 ± 0.016 | 0.459 ± 0.035 | 0.751 ± 0.015 | 0.493 ± 0.052 | 0.565 ± 0.012 | 0.525 ± 0.016 | 0.582 ± 0.010 | 0.787 ± 0.006 |
| **Gene PCC** | 0.147 ± 0.022 | 0.328 ± 0.027 | 0.227 ± 0.019 | 0.226 ± 0.028 | 0.005 ± 0.009 | 0.018 ± 0.014 | 0.170 ± 0.008 | 0.164 ± 0.012 |
| **Top 50 PCC** | 0.401 ± 0.037 | 0.640 ± 0.024 | 0.451 ± 0.031 | 0.501 ± 0.026 | 0.105 ± 0.013 | 0.089 ± 0.011 | 0.410 ± 0. 022 | 0.371 ± 0.010 |
| **Patch SC** | 0.354 ± 0.027 | 0.332 ± 0.034 | 0.456 ± 0.022 | 0.480 ± 0.011 | 0.447 ± 0.023 | 0.420 ± 0.016 | 0.475 ± 0.012 | 0. 665 ± 0.012 |
| **Gene SC** | 0.043 ± 0.021 | 0.282 ± 0.026 | 0.247 ± 0.021 | 0.225 ± 0.026 | 0.004 ± 0.007 | 0.013 ± 0.012 | 0.164 ± 0.007 | 0.159 ± 0.016 |
| **Top 50 SC** | 0.394 ± 0.03 | 0.555 ± 0.025 | 0.501 ± 0.038 | 0.530 ± 0.026 | 0.106 ± 0.012 | 0.086 ± 0.010 | 0.411 ± 0.017 | 0.3585 ± 0.02 |
