# Supplementary material for: Spatially guided translation from histology images to transcriptomic profiles using foundation model-driven contrastive learning
Source: PLoS Comput Biol. 2026 Sep 8;22(9):e1014762. doi: 10.1371/journal.pcbi.1014762 (PMC13568522; doi:10.1371/journal.pcbi.1014762)
Supplement: Supplementary file 5 [file pcbi.1014762.s005.docx]

**S3 Table.** LOOCV results for FOCST by patient_ID with cSCC dataset. PCC reported at the patch, gene, and top50 gene level.

|  | **P2** | **P5** | **P9** | **P10** |
| --- | --- | --- | --- | --- |
| **Patch PCC** | 0.362 ± 0.018 | 0.514 ± 0.034 | 0.453 ± 0.023 | 0.529 ± 0.0083 |
| **Gene PCC** | 0.244 ± 0.015 | 0.143 ± 0.027 | 0.210 ± 0.016 | 0.182 ± 0.032 |
| **Top 50 PCC** | 0.403 ± 0.024 | 0.170 ± 0.039 | 0.314 ± 0.018 | 0.294 ± 0.046 |
| **Patch SC** | 0.362 ± 0.098 | 0.394 ± 0.021 | 0.452 ± 0.029 | 0.471 ± 0.035 |
| **Gene SC** | 0.275 ± 0.015 | 0.117 ± 0.022 | 0.227 ± 0.028 | 0.184 ± 0.019 |
| **Top 50 SC** | 0.490 ± 0.028 | 0.221 ± 0.030 | 0.353 ± 0.030 | 0.293 ± 0.026 |
