# Supplementary material for: Spatially guided translation from histology images to transcriptomic profiles using foundation model-driven contrastive learning
Source: PLoS Comput Biol. 2026 Sep 8;22(9):e1014762. doi: 10.1371/journal.pcbi.1014762 (PMC13568522; doi:10.1371/journal.pcbi.1014762)
Supplement: Supplementary file 6 [file pcbi.1014762.s006.docx]

**S4 Table.** Ablation Study of Temperature setting. Mean PCC values are calculated for the HER2-positive dataset for the patch level, gene level, and the top 50 highest correlated genes. Bolded represents the lowest performing ablation experiment.

| **Method** | **Patch** | **Gene** | **Top 50** |
| --- | --- | --- | --- |
| **FOCST (Temp = 1)** | **0.648** | **0.139** | **0.388** |
| No Smoothing | 0.536 | 0.106 | 0.285 |
| Temp = 0.5 | 0.604 | 0.121 | 0.347 |
| Temp = 2 | 0.628 | 0.124 | 0.363 |
