# Supplementary material for: Spatially guided translation from histology images to transcriptomic profiles using foundation model-driven contrastive learning
Source: PLoS Comput Biol. 2026 Sep 8;22(9):e1014762. doi: 10.1371/journal.pcbi.1014762 (PMC13568522; doi:10.1371/journal.pcbi.1014762)
Supplement: Supplementary file 7 [file pcbi.1014762.s007.docx]

**S5 Table.** Sensitivity study of nearest neighbor values. Mean PCC values are calculated for the HER2-positive dataset for the patch level, gene level, and the top 50 highest correlated genes. Bolded represents the best performing selection.

| **Method** | **Patch** | **Gene** | **Top 50** |
| --- | --- | --- | --- |
| **FOCST (n = 8)** | **0.648** | **0.139** | **0.388** |
| n = 4 | 0.639 | 0.129 | 0.395 |
| n = 16 | 0.635 | 0.128 | 0.382 |
