# Supplementary material for: Spatially guided translation from histology images to transcriptomic profiles using foundation model-driven contrastive learning
Source: PLoS Comput Biol. 2026 Sep 8;22(9):e1014762. doi: 10.1371/journal.pcbi.1014762 (PMC13568522; doi:10.1371/journal.pcbi.1014762)
Supplement: Supplementary file 8 [file pcbi.1014762.s008.docx]

**S6 Table.** Sensitivity study of α and β values. Mean PCC values are calculated for the HER2-positive dataset for the patch level, gene level, and the top 50 highest correlated genes. Bolded represents the best performing selection.

| **Method** | **Patch** | **Gene** | **Top 50** |
| --- | --- | --- | --- |
| **FOCST (α = 0.1, β = 0.25)** | **0.648** | **0.139** | **0.388** |
| α = 0.2, β = 0.25 | 0.635 | 0.129 | 0.388 |
| α = 0.05, β = 0.25 | 0.637 | 0.127 | 0.380 |
| α = 0.1, β = 0.5 | 0.637 | 0.130 | 0.382 |
| α = 0.1, β = 0.1 | 0.634 | 0.128 | 0.381 |
