# Supplementary material for: Spatially guided translation from histology images to transcriptomic profiles using foundation model-driven contrastive learning
Source: PLoS Comput Biol. 2026 Sep 8;22(9):e1014762. doi: 10.1371/journal.pcbi.1014762 (PMC13568522; doi:10.1371/journal.pcbi.1014762)
Supplement: Supplementary file 9 [file pcbi.1014762.s009.docx]

**S7 Table**. FOCST pipeline with Prov-Gigapath backbone as image encoder. Mean PCC values are calculated for the HER2-positive dataset for the patch level, gene level, and the top 50 highest correlated genes. Bolded represents highest scoring.

| Test Image (# of spots) | Patch PCC | Gene PCC | Top 50 PCC |
| --- | --- | --- | --- |
| A1 (346) | 0.1991 | 0.0213 | 0.1525 |
| B1 (295) | 0.3633 | **0.3558** | **0.6636** |
| C1 (176) | **0.5572** | 0.1375 | 0.4000 |
| D1 (306) | 0.4806 | 0.1542 | 0.4223 |
| E1 (587) | 0.4111 | -0.0158 | -0.0986 |
| F1 (691) | 0.5368 | -0.0205 | -0.0722 |
| G2 (467) | 0.5051 | 0.1406 | 0.4101 |
| H1 (613) | 0.3275 | 0.1535 | 0.3761 |
