# Supplementary material for: Spatially guided translation from histology images to transcriptomic profiles using foundation model-driven contrastive learning
Source: PLoS Comput Biol. 2026 Sep 8;22(9):e1014762. doi: 10.1371/journal.pcbi.1014762 (PMC13568522; doi:10.1371/journal.pcbi.1014762)
Supplement: Supplementary file 10 [file pcbi.1014762.s010.docx]

**S8 Table :** Preprocessing checklist

| **Dataset** | **Gene List Preprocess** | **Expression Preprocess** | **Image Preprocess** |
| --- | --- | --- | --- |
| **HER2-positive, cSCC, Lung** | - Identify top 1000 HVGs across all samples / spots - Discard genes that are expressed in less than 1000 spots | - N/A | - N/A |
| **Yale (FOCST input)** | - N/A | - N/A | - LuminosityStandardizer - StainNormalizer with Vahadane - Generate image patches of (224 pixels x 224 pixels) - Filter out patches where ratio of black region (tissue) to total pixel is less than 75% |
| **Treatment Response Classifier** | - Genes expressed in less than 3 cells | - Cells expressing fewer than 100 genes are removed | - LuminosityStandardizer - StainNormalizer with Vahadane - Generate image patches of (224 pixels x 224 pixels) - Filter out patches where ratio of black region (tissue) to total pixel is less than 75% |
